# Sex-based differences in placental DNA methylation profiles related to gestational age: an NIH ECHO meta-analysis

**DOI:** 10.1080/15592294.2023.2179726

**Published:** 2023-02-25

**Authors:** Catherine M. Bulka, Todd M. Everson, Amber A. Burt, Carmen J. Marsit, Margaret R. Karagas, Kristen E. Boyle, Sierra Niemiec, Katerina Kechris, Elizabeth J. Davidson, Ivana V. Yang, Jason I. Feinberg, Heather E. Volk, Christine Ladd-Acosta, Carrie V. Breton, T. Michael O’Shea, Rebecca C. Fry

**Affiliations:** a Department of Environmental Sciences and Engineering, University of North Carolina at Chapel Hill, Chapel Hill, NC, USA; b College of Public Health, University of South Florida, Tampa, FL, USA; c Gangarosa Department of Environmental Health, Emory University Rollins School of Public Health, Atlanta, GA, USA; d Department of Epidemiology, Geisel School of Medicine at Dartmouth, Hanover, NH, USA; e Section of Nutrition, Department of Pediatrics, University of Colorado Anschutz Medical Campus, Aurora, CO, USA; f Colorado School of Public Health, The Lifecourse Epidemiology of Adiposity and Diabetes (LEAD) Center, Aurora, CO, USA; g Department of Biostatistics & Informatics, Colorado School of Public Health, Aurora, CO, USA; h Department of Medicine, University of Colorado School of Medicine, Aurora, CO, USA; i Department of Mental Health, Johns Hopkins Bloomberg School of Public Health, Baltimore, ML, USA; j Department of Epidemiology, Johns Hopkins Bloomberg School of Public Health, Baltimore, ML, USA; k Department of Population and Public Health Sciences, University of Southern California, Los Angeles, CA, USA; l Department of Pediatrics, School of Medicine, University of North Carolina at Chapel Hill, Chapel Hill, NC, USA; m Institute for Environmental Health Solutions, University of North Carolina at Chapel Hill, Chapel Hill, NC, USA; n Curriculum in Toxicology and Environmental Medicine, University of North Carolina at Chapel Hill, Chapel Hill, NC, USA

**Keywords:** Placenta, gestational age, DNA methylation, sex differences

## Abstract

The placenta undergoes many changes throughout gestation to support the evolving needs of the foetus. There is also a growing appreciation that male and female foetuses develop differently *in utero*, with unique epigenetic changes in placental tissue. Here, we report meta-analysed sex-specific associations between gestational age and placental DNA methylation from four cohorts in the National Institutes of Health (NIH) Environmental influences on Child Health Outcomes (ECHO) Programme (355 females/419 males, gestational ages 23–42 weeks). We identified 407 cytosine-guanine dinucleotides (CpGs) in females and 794 in males where placental methylation levels were associated with gestational age. After cell-type adjustment, 55 CpGs in females and 826 in males were significant. These were enriched for biological processes critical to the immune system in females and transmembrane transport in males. Our findings are distinct between the sexes: in females, associations with gestational age are largely explained by differences in placental cellular composition, whereas in males, gestational age is directly associated with numerous alterations in methylation levels.

## Introduction

The placenta is a key regulator of foetal growth and development. By remodelling uterine spiral arteries, transporting oxygen and nutrients, removing waste products, releasing hormones, promoting maternal immune tolerance, and providing protection from xenobiotics, the placenta plays a critical role in establishing and maintaining a healthy pregnancy [1,2]. Abnormalities of the placenta have been proposed to underlie a myriad of pregnancy complications, including preterm labour [3], preeclampsia [4], gestational diabetes [5], placenta accreta [6], placental abruption [7], and intrauterine growth restriction [8], together affecting as many as 1 in 10 pregnancies. Placental structure and characteristics can have a lasting impact on the health of the offspring that extends beyond pregnancy. For example, measures of gross placental structure, including placental weight, thickness, and diameter, are predictive of growth during childhood even after considering their direct influence on size at birth [9]. To gain fundamental insights into the placenta’s role in immediate and long-term health, researchers are characterizing its molecular landscape. Although studies have shown that gene regulation of the placenta evolves dramatically throughout gestation [10–13], examining molecular regulators of gene expression is necessary to more comprehensively describe biological pathways involved in normal human development and disease pathogenesis.

One important regulator of gene expression is DNA methylation, which refers to the addition of a methyl group to the DNA strand. DNA methylation is most commonly measured at cytosine-guanine dinucleotides (CpGs), a sequence that is relatively rare across the genome [14]. Areas where CpGs are comparatively dense and cluster together are known as CpG islands; in humans, an estimated 70% of genes have a CpG island in their promoter region [15]. Increasing levels of methylation at a promoter-associated CpG island tend to correspond to transcriptional repression [16]. In contrast, increased methylation within the gene body is often positively associated with expression [17, 18]. Although these general patterns may be more complex in the human placenta [19], describing how CpG methylation relates to the length of gestation may provide insights into foetal growth and development as well as the molecular processes underlying links between intrauterine exposures and later-in-life health.

Several prior studies have examined the associations of gestational age with CpG methylation in cord blood [20–25] and placental tissue [26–28]. However, despite the fact that female and male foetuses exhibit distinct patterns of growth and development, no study to date has considered whether the relationship between gestational age and DNA methylation differs by foetal sex. Beginning in the first trimester, there are measurable differences in foetal size by sex, with males having greater crown-rump length in addition to larger abdominal and head circumferences [29]. Despite their size advantage, male foetuses are generally more susceptible to pregnancy complications [30, 31]. After birth, male infants are also more likely than females to exhibit morbidities, including respiratory distress and neurological impairments [32–35]; it has been posited that the pathogenesis of these conditions originates *in utero* and is influenced by the placenta [36, 37].

The placenta is a sexually dimorphic organ. Female placentas exhibit global upregulation of autosomal genes, particularly for genes involved in immune regulation [38]. In addition, variation in the placental methylome by foetal sex has been observed at an appreciable number of autosomal CpGs, for which males tend to have higher levels of methylation [27, 39, 40]. Given such striking patterns, it is possible that important age-related alterations of the placenta could be obscured when not considering biological sex. In the present work, we investigate sex-specific patterns of placental CpG methylation in relation to gestational age. We provide results from four birth cohorts in the National Institutes of Health (NIH) Environmental influences on Child Health Outcomes (ECHO) Programme that we meta-analysed to detect robust differential methylation signals. We conducted analyses that considered associations of placental CpG methylation with gestational age directly, as well as associations corrected for differences in placental cell composition. We explored whether associations of gestational age and placental CpG methylation were distinct between females and males. Finally, we performed enrichment analyses of identified genes to contextualize the biological processes implicated by our findings.

## Methods

### Study population

Birth cohorts in the NIH ECHO programme were invited to participate in the present analysis if they had placental DNA methylation data available. Based on this criteria, four cohorts – the Early Autism Risk Longitudinal Investigation (EARLI), Extremely Low Gestational Age Newborns (ELGAN), Healthy Start, and the New Hampshire Birth Cohort Study (NHBCS) – were included in this study. Healthy Start and the NHBCS were general birth cohorts, whereas EARLI enrolled infants with a sibling affected by autism spectrum disorder and ELGAN enrolled infants born extremely preterm (<28 weeks’ gestation). Only singleton births were eligible for study inclusion and all had complete data on gestational age and assigned sex at birth. Placental tissue DNA samples that failed cohort-specific quality control procedures were excluded (as described in further detail in the **Supplementary Materials**). In addition, cohorts were instructed to exclude samples with potentially erroneous data, including gestational ages recorded as <22 or >42 weeks and any samples with discrepancies between their reported and methylation-predicted sex (**Supplemental Figure 1**). Sex prediction was ascertained using the *minfi* package by comparing the median values of probes mapped to the X and Y chromosomes [41], which disagreed with the reported sex for 4 ELGAN and 2 Healthy Start samples (**Supplemental Figure 1**). Additional details regarding cohort eligibility criteria and participant characteristics can be found in the **Supplementary Materials** and **Supplemental Table 1**. Each cohort received ethical approval from local institutional review boards and obtained informed parental consent for all participants. Each cohort received ethical approval from local institutional review boards and obtained informed parental consent for all participants.

### Gestational age ascertainment

Gestational ages were measured differently in each cohort. In EARLI, gestational ages were obtained from the labour and delivery nurse present at delivery when possible; otherwise, gestational age was calculated as the difference between the date of birth and the estimated delivery date minus 40 weeks (280 days). In ELGAN, gestational ages were estimated based on a hierarchy of the quality of available information with dates of embryo retrieval or intrauterine insemination or foetal ultrasound dating considered most accurate, followed by estimates based on last menstrual period date. In Healthy Start, gestational age at birth was obtained from the delivery medical record. Finally, in the NHBCS, gestational age was determined by foetal ultrasounds occurring between 11 and 13 weeks when possible; otherwise, it was estimated using the last menstrual period date. Gestational age was analysed in completed decimal weeks (e.g., 37 weeks and 2 days was recorded as 37.3 weeks).

### Methylation measurements

DNA methylation from placental tissue was measured using either the Illumina Infinium HumanMethylation450K or the MethylationEPIC (850 K) platform. Each cohort conducted their own quality control and normalization of DNA methylation data, as detailed in the **Supplementary Materials**. Cohorts retained all CpGs that passed quality control and corrected for technical aspects related to processing (i.e., batch effects) in their data using *ComBat* (**Supplemental Table 2**) [42]. Quality control checks revealed that several cohort-specific genomic inflation factors exceeded 1.25 (as shown in **Supplemental Table 3** and **Supplemental Figure 2**). Thus, we implemented a Bayesian method, BACON, to estimate empirical null distributions to the data [43]. Doing so resulted in markedly lower inflation values with a maximum value of 1.24. The BACON-corrected model coefficients and corresponding standard errors were subsequently used for all meta-analyses.

### Coordinated epigenome-wide association studies and estimation of placental cell-type composition

Each cohort performed independent epigenome-wide association studies (EWASs) according to a pre-specified analytic plan. For each CpG site, placental methylation was reported as the average β-value, ranging from 0 (unmethylated) to 1 (fully methylated). Two sex-stratified models were fit using the R package *limma* [44]: 1) an unadjusted model with placental methylation β-values as the dependent variable and gestational age (continuous) as the independent variable; and 2) an adjusted model that included estimated proportions of trophoblasts, syncytiotrophoblast, stromal, Hofbauer, endothelial, and nucleated red blood cells as model covariates [45]. By comparing the coefficients for gestational age from the two models within each sex strata, we can make an indirect assessment about the extent to which increasing gestational age may be altering the cellular composition of the placenta [46]. Large differences between the unadjusted and cell-type adjusted coefficients are proposed to represent CpG methylation signals that are not independently correlated with the amount of time spent *in utero* but rather are primarily driven by differences in cell-type proportions.

Proportions of the placental cell types were estimated using a reference-based deconvolution method via the R package *planet* [45]. The *planet* algorithm was developed by characterizing the methylomes of placentas collected from terminated and healthy term pregnancies and therefore requires that users specify whether their samples were collected during the first or third trimester. For our analyses, we applied this approach as designed for all third trimester samples (≥28 weeks gestation); for any second trimester samples (<28 weeks gestation), we interpolated cell-type proportions by simply averaging across first and third trimester estimates per the *planet* authors’ recommendations (V. Yuan and W. Robinson, personal communication, 17 February 2021). Heatmaps of the estimated placental cell-type proportions were produced by each cohort using R package *pheatmap* [47], with hierarchical clustering performed using Ward’s method and annotations for infant sex and gestational age [48].

### Meta-analyses

Prior to meta-analyses, we removed probes that were not shared across the 450 K and EPIC arrays and those that mapped to sex chromosomes, targeted non-CpG sites, were associated with single-nucleotide polymorphisms, or were previously identified as cross-hybridizing (**Supplemental Figure 1**) [49]. We also filtered out CpGs not retained in at least two of the study cohorts, for a maximum total of 354,869 probes for the analyses.

Next, for each of the four models (females, females with cell-type adjustment, males, males with cell-type adjustment), we calculated cohort-specific genomic inflation statistics and generated QQ plots to assess test statistic inflation. We used BACON, a Bayesian method to evaluate and control for test statistic bias and inflation that was specifically developed to deal with inflation in EWASs [43].

We then performed inverse-variance weighted fixed-effect meta-analyses using the R package *metafor* [50]. Genome-wide significance was based on an FDR correction of q < 0.05 [51]. We explored between-study variability by calculating the I^2^ statistic, which describes the extent to which each cohort’s results were consistent. We considered I^2^ values ≤50% to reflect homogeneity across studies. For probes with I^2^ values <50%, we re-ran meta-analyses using random-effects models fitted with restricted maximum likelihood, replacing fixed-effect estimates with their random-effects counterparts to better account for between-study heterogeneity [52]. In addition, we conducted leave-one-out analyses on all FDR-significant probes, in which we performed the overall meta-analysis repeatedly, excluding one of the four cohorts in each run, allowing us to identify whether any single cohort was overly influential.

### Testing for sex-specific associations of gestational age with placental CpG methylation

To formally test whether relationships between gestational age and placental CpG methylation differed by sex, we performed heterogeneity testing on probes that reached FDR-significance. These analyses were focused on results from the cell-type–adjusted models to control for possible sex-based differences in the cellular composition of the placenta. Specifically, we used two-sample Z-tests to test for equality between the meta-analysed, BACON-corrected, cell-type–adjusted regression coefficients from males and females [53]. This approach can be considered analogous to modelling gestational age in an interaction cross-product term with infant sex but is less biased in the event that any associations between placental cell types and methylation at individual CpGs are sex-dependent [54]. To identify whether gestational age – placental CpG methylation associations significantly differed between males and females, the FDR approach was implemented to account for multiple testing.

### CpG annotation and pathway analyses

We annotated CpG sites to genes using the Infinium HumanMethylation450K manifest file provided by Illumina, which references Genome Build 37 (hg19) [55], and we verified the annotations for Bonferroni-significant CpGs in the UCSC Genome Browser [56]. We then performed GO pathway analyses using the gometh function from the *missMethyl* R package to identify potential developmental mechanisms linking gestational age with placental CpG methylation [57]. This function maps CpGs to genes and performs hypergeometric tests that account for the differing number of probes within each gene. We focused these pathway analyses on CpGs that were significantly associated with gestational age within each sex strata at q < 0.05 after adjusting for cell-type heterogeneity. For each sex, the top 25 GO terms (all p < 0.01) for biological processes were consolidated and summarized using REVIGO, which removes redundant concepts [58]. The results from REVIGO were visualized using TreeMaps that show clusters of GO terms as rectangles; the colours depict related clustered GO terms and the size of the rectangles reflect -log_10_(p-values).

## Results

### Study population

Our analyses included data from four demographically diverse United States-based birth cohort studies that are part of the ECHO Programme: (1) the Early Autism Risk Longitudinal Investigation (EARLI), which enrolled infants with a sibling affected by autism spectrum disorder, (2) the Extremely Low Gestational Age Newborns (ELGAN) study, which enrolled infants born extremely preterm (<28 weeks’ gestation), (3) the Healthy Start study, and (4) the New Hampshire Birth Cohort Study (NHBCS), which were both general birth cohorts. Of the four cohorts, EARLI and NHBCS profiled placental CpG methylation using the Infinium HumanMethylation450K BeadChip, whereas Healthy Start and ELGAN used the MethylationEPIC BeadChip (850 K). The cohorts derived gestational age from multiple sources, including medical records using ultrasound estimation, last menstrual period date, or, for pregnancies conceived by assisted reproductive technology, dates of embryo retrieval or intrauterine insemination. Gestational ages ranged from 23 to 42 weeks (Table 1). Hierarchically clustered heatmaps of the placental cell-type proportions estimated by each cohort are provided in **Supplemental Figures 3–6**. The six cell types estimated by *planet* were clearly differentiated with syncytiotrophoblasts comprising the largest proportion and trophoblasts comprising the smallest proportion. Within the ELGAN cohort (**Supplemental Figure 4**), which was composed of infants born extremely preterm (<28 weeks gestation), there were no apparent patterns in placental cell-type proportions by infant sex. However, within the other cohorts (**Supplemental Figures 3, 5**, and **6**), clustering revealed more closeness in cell-type proportions by infant sex than by gestational age.

**Table 1.** A summary of key information from participating cohorts.

	Females			Males			Gestational Age Data Source	Methylation Array
Cohort	N	Gestational Age (weeks)		N	Gestational Age (weeks)			
		Median	Range		Median	Range		
Early Autism Risk Longitudinal Investigation (EARLI)	37	40.0	36.6–41.6	54	39.3	36.3–41.6	Reported by the labour and delivery nurse present at delivery; if unavailable, calculated as: (date of birth – (estimated delivery date – 280))/7	450 K
Extremely Low Gestational Age Newborns (ELGAN)	112	26.4	23.0–27.9	130	26.0	23.0–27.9	Determined by foetal ultrasound or dates of embryo retrieval or intrauterine insemination; if unavailable, calculated as: (date of birth – last menstrual period date)/7	EPIC
Healthy Start	47	39.7	36.1–41.7	51	39.3	35.1–41.1	Obtained from the medical record at delivery	EPIC
New Hampshire Birth Cohort Study (NHBCS)	159	39.0	31.8–41.9	184	39.0	31.0–41.9	Determined by foetal ultrasound; if unavailable, calculated as: (date of birth – last menstrual period date)/7	450 K
*Overall*	355	36.3	23.0–42.0	419	35.9	23.0–41.9		

### Sex-specific meta-analyses of gestational age

**Figure 1.**
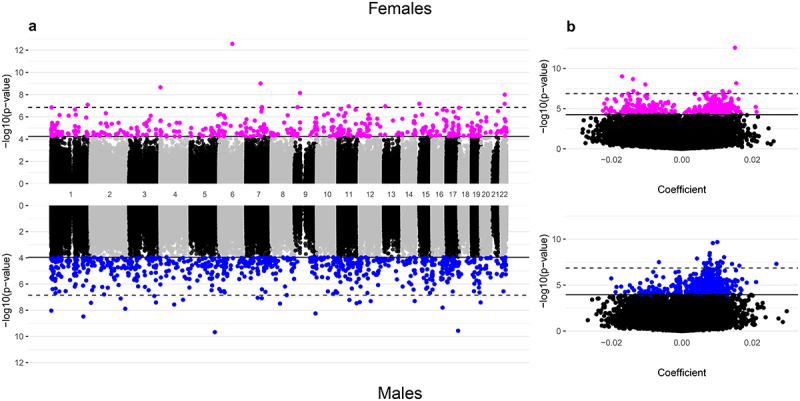
Manhattan and volcano plots of the inverse-variance fixed meta-analysis results for gestational age and placental CpG methylation among EARLI, ELGAN, healthy start, and NHBCS.

**Figure 2.**
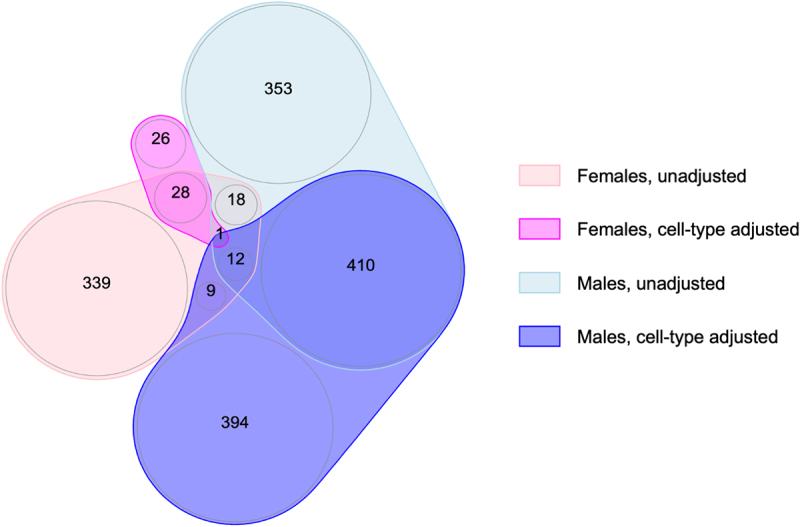
Overlap of FDR-significant CpGs across the meta-analyses of gestational age and placental methylation among females and males from EARLI, ELGAN, healthy start, and NHBCS, with and without cell-type adjustment.

We identified a total of 1201 CpGs associated with gestational age. Specifically, 407 CpGs displayed methylation associated with gestational age among females and 794 CpGs associated with gestational age among males at q < 0.05 (Table 2 and Figure 1). Of the false discovery rate (FDR)-significant CpGs, we identified only 19 that were common between males and females (Figure 2 and **Supplemental Table 4**). Within each sex, most of the FDR-significant associations survived the leave-one-out analyses (Table 2), suggesting they were not driven by a single cohort. In addition, the majority of FDR-significant CpGs (99.8% in females and 99.7% in males) had I^2^ values ≤50%, indicating that the results were highly consistent across the four cohorts. We highlight the sex-specific top 10 FDR-significant CpGs from the unadjusted models in Table 3 but have also provided results for all CpGs in **Supplemental Table 5**. The most significant association observed in the unadjusted models of gestational age in females was for cg06677013, located in the gene body of *CYB5R4* (Table 3); specifically, each additional week of gestation corresponded to a 0.015 (p-value = 2.71 × 10^−13^) increase in placental methylation β-values at this specific probe, a relationship that persisted even when single cohorts were omitted in the leave-one-out analysis. In males, the top hit was for cg05169312 (*GEMIN5*), for which a 1-week increase in gestational age in males corresponded to a 0.010 (p-value = 2.12 × 10^−10^) increase in placental methylation levels, which also did not appear to be driven by any one cohort (Table 3).

**A**. Manhattan plots of placental CpGs associated with gestational age among females (N = 355; top) and males (N = 419; bottom); **B**. Volcano plots of placental CpGs associated with gestational age among females (top) and males (bottom). In the Manhattan plots, the x-axes represent genomic locations. In the volcano plots, the x-axes show the estimated mean difference in methylation β-values for a 1-week increase in gestational age. Y-axes for both plots show -log_10_(p-values) with the solid horizontal black line indicating the false discovery rate (q < 0.05) and the dashed horizontal black line indicating the more conservative Bonferroni threshold (1.41 × 10^−7^) for statistical significance.

Female-specific results without cell-type adjustment are shown in light pink and with cell-type adjustment are shown in dark pink; male-specific results without cell-type adjustment are shown in light blue and with cell-type adjustment are shown in dark blue.

**Table 2.** A summary of the number of identified CpG sites from each EWAS meta-analysis of gestational age.

	Females (*N* = 355)		Males (*N* = 419)	
Model	FDR-significant CpGs	Survived leave-one-out	FDR-significant CpGs	Survived leave-one-out
Unadjusted	407	382	794	598
Cell-type–adjusted	55	3	826	679

The false discovery rate (FDR) threshold for statistical significance was q < 0.05. Leave-one-out refers to performing the meta-analyses excluding one cohort each time. When any single cohort was omitted, ‘survival’ was defined as: (1) having a p-value < 0.05, (2) maintaining an association in the same direction as the overall meta-analysis; (3) maintaining a magnitude of association within 50% of the overall meta-analysis. EWAS, epigenome-wide association study.

**Table 3.** Top 10 FDR-significant CpGs for the meta-analysis of gestational age and placental methylation among females and males from the EARLI, ELGAN, Healthy Start, and NHBCS cohorts.

Probe (Gene)	Coefficient (95% CI)	*Q*-value	Heterogeneity *I*^2^ (*P*-value)	Direction in Each Cohort	Survived leave-one-out
**Females (*N = *355)**					
cg06677013 (*CYB5R4*)	0.015 (0.011, 0.019)	9.60E-08	0.0 (0.39)	++++	Yes
cg03094728 (*AKAP9*)	−0.017 (−0.022, −0.012)	1.76E-04	41.2 (0.18)	– ?	Yes
cg23657718 (*AX748388^+^*)	−0.014 (−0.018, −0.009)	2.58E-04	34.4 (0.21)	– -	Yes
cg15271616 (*RUSC2*)	0.015 (0.010, 0.021)	6.23E-04	0.0 (0.65)	++++	Yes
cg10649156 (*RPL3^+^*)	−0.010 (−0.014, −0.007)	7.12E-04	0.0 (0.68)	– -	Yes
cg09290175 (*RPL3^+^*)	−0.014 (−0.019, −0.009)	3.37E-03	0.0 (0.60)	– -	Yes
cg22821677 (*LOC646214^+^*)	0.012 (0.007, 0.016)	3.57E-03	0.0 (0.75)	++++	Yes
cg10821976 (*TSNAX-DISC1*)	0.013 (0.009, 0.018)	3.78E-03	24.2 (0.27)	-+++	Yes
cg03982074 (*MUC12*^+^)	−0.013 (−0.017, −0.008)	9.60E-08	48.5 (0.12)	– -	Yes
cg08995609 (*RIN1*)	0.007 (0.005, 0.010)	2.58E-04	24.6 (0.26)	++++	Yes
**Males (*N* = 419)**					
cg05169312 (*GEMIN5*)	0.010 (0.007, 0.013)	4.82E-05	0.0 (0.43)	++++	Yes
cg05875982 (*PRCD*)	0.009 (0.006, 0.012)	4.82E-05	41.9 (0.16)	++++	Yes
cg12447744 (*MDM4*)	0.008 (0.005, 0.011)	3.94E-04	0.0 (0.52)	++++	Yes
cg14462758 (*EXOSC2*)	0.008 (0.005, 0.011)	5.07E-04	0.0 (0.80)	++++	Yes
cg20285026 (*PRKCZ*)	0.012 (0.008, 0.016)	6.62E-04	0.0 (0.49)	++++	Yes
cg11373252 (*NHEJ1^+^*)	0.004 (0.003, 0.006)	7.67E-04	0.0 (0.53)	++++	Yes
cg08827358 (*BEAN1*)	0.008 (0.005, 0.011)	7.99E-04	0.0 (0.54)	++++	Yes
cg04503912 (*DSPP^+^*)	0.011 (0.007, 0.015)	1.05E-03	0.0 (0.51)	++++	Yes
cg07642463 (*ZNF264*)	0.010 (0.006, 0.014)	5.07E-04	0.0 (0.43)	++++	Yes
cg07954075 (*LOC107984507^+^*)	0.006 (0.004, 0.009)	6.62E-04	24.2 (0.27)	++++	Yes

Coefficients correspond to the expected change in placental DNA methylation per additional week of gestation. Gene annotations were verified in the UCSC Genome Browser (hg19) with *^+^* indicating the closest gene. The direction of coefficients is ordered by cohort as follows: EARLI, ELGAN, Healthy Start, NHBCS. A ‘?’ mark indicates the respective cohort was missing methylation data for a given probe.

### Sex-specific meta-analyses of gestational age after cell-type adjustment

**Figure 3.**
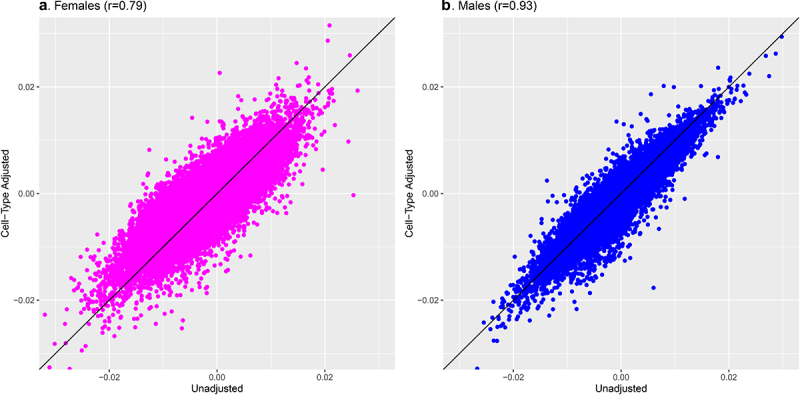
Comparison of meta-analysis model coefficients for gestational age and placental CpG methylation among EARLI, ELGAN, Healthy Start, and NHBCS before and after cell-type adjustment.

Adjusting for proportions of placental cell types attenuated the number of gestational age–associated CpGs identified among females from 407 to 55 (Table 2). Of these, all had I^2^ values less than 50%, but only 3 survived the leave-one-out analyses, suggesting the associations observed were not robust. Among males, the number of FDR-significant CpGs associated with gestational age after adjusting for cell-type heterogeneity remained high, with 826 FDR-significant CpGs in the cell-type–adjusted model versus 794 FDR-significant CpGs in the unadjusted model. For males, the cell–type adjusted associations appeared remarkably stable: all of the 826 associations deemed FDR-significant had I^2^ values below 50% and the majority (679 of 826, 82.2%) survived the leave-one-out analyses. Comparing the magnitude of model coefficients from the unadjusted and cell-type–adjusted models revealed generally positive correlations (Figure 3), although they were more strongly correlated among male-derived placentas (r = 0.93 in males; r = 0.79 in females). This suggests that gestational age has a stronger and more direct relationship with the placental methylome of males compared with females, for whom associations appear to be largely explained by differences in cell-type composition.

**A**. Scatter plot of unadjusted and cell-type adjusted model coefficients for a 1-week increase in gestational age among females (N = 355); **B**. Scatter plot of unadjusted and cell-type adjusted model coefficients for a 1-week increase in gestational age among males (N = 419).

**Figure 4.**
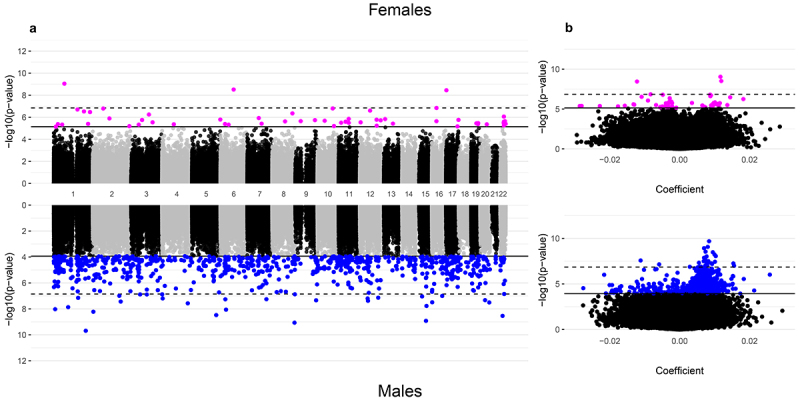
Manhattan and volcano plots of the inverse-variance fixed meta-analysis results for gestational age and placental CpG methylation after cell-type adjustment among EARLI, ELGAN, healthy start, and NHBCS.

The 55 CpGs among females and 826 CpGs among males associated with gestational age after adjustment for placental cell-type composition were annotated to 41 genes and 538 genes, respectively. For females, negative and positive associations between gestational age and placental CpG methylation levels were observed in approximately equal numbers (Figure 4). However, for males, a 1-week increase in gestational age at birth exhibited a predominance of positive associations with placental methylation levels (86% of FDR-significant CpGs in males; Figure 4). We provide results for the top 10 FDR-significant CpGs after cell-type adjustment in Table 4 while relationships for all CpGs, along with detailed annotations, are included in **Supplemental Table 6**.

**A**. Manhattan plots of placental CpGs associated with gestational age after adjustment for cell-type composition among females (N = 355; top) and males (N = 419; bottom); **B**. Volcano plots of placental CpGs associated with gestational age after adjustment for cell-type composition among females (top) and males (bottom). In the Manhattan plots, the x-axes represent genomic locations, whereas in the volcano plots, the x-axes show the estimated mean difference in methylation β-values for a 1-week increase in gestational age. Y-axes for both plots show -log_10_(p-values) with the solid horizontal black line indicating the false discovery rate (q < 0.05) and the dashed horizontal black line indicating the more conservative Bonferroni threshold (1.41 × 10^−7^) for statistical significance.

**Table 4.** Top 10 FDR-significant CpGs for the meta-analysis of gestational age and placental methylation with adjustment for cell-type proportions among females and males from the EARLI, ELGAN, healthy start, and NHBCS cohorts.

Probe (Gene)	Coefficient (95% CI)		*Q*-value	Heterogeneity *I*^2^ (*P*-value)		Direction in Each Cohort	Survived leave-one-out
**Females (*N = *355)**							
cg11650925 (*GNG12-AS1*)	0.012 (0.008, 0.015)	3.18E-04			21.5 (0.28)	++++	No
cg06677013 (*CYB5R4*)	0.012 (0.008, 0.016)	4.19E-04			36.8 (0.19)	++++	No
cg15030415 (*RAP1GAP2*)	−0.012 (−0.016, −0.008)	4.19E-04			35.2 (0.20)	– -	No
cg04732596 (*MEIS1*)	−0.005 (−0.007, −0.003)	9.63E-03			0.0 (0.43)	– -	No
cg10113589 (*OPALIN*)	0.009 (0.005, 0.012)	9.63E-03			41.3 (0.16)	++++	No
cg26709300 (*YPEL3*)	−0.008 (−0.011, −0.005)	9.63E-03			38.7 (0.18)	– -	No
cg02959609 (*C1orf230*)	0.009 (0.006, 0.012)	9.93E-03			15.6 (0.31)	++++	No
cg15175129 (*MSRB3*)	0.009 (0.005, 0.012)	1.10E-02			43.8 (0.15)	++++	No
cg12617598 (*CDC73*)	−0.011 (−0.015, −0.007)	1.16E-02			26.3 (0.25)	– -	No
cg10821976 (*TSNAX-DISC1*)	0.014 (0.009, 0.02)	1.20E-02			0.0 (0.62)	++++	No
**Males (*N* = 419)**							
cg12447744 (*MDM4*)	0.008 (0.006, 0.011)	7.32E-05			40.5 (0.17)	++++	Yes
cg12134349 (*SLC45A4; LOC105375787*)	0.008 (0.005, 0.011)	1.42E-04			27.9 (0.24)	++++	Yes
cg23323879 (*CILP^+^*)	0.007 (0.005, 0.009)	1.42E-04			0.0 (0.82)	++++	Yes
cg05169312 (*GEMIN5*)	0.010 (0.006, 0.013)	2.37E-04			0.0 (0.53)	++++	Yes
cg17402397 (*SMTN*)	0.007 (0.005, 0.010)	2.37E-04			0.0 (0.76)	++++	Yes
cg09753772 (*RPS7^+^*)	0.010 (0.007, 0.013)	3.57E-04			1.3 (0.39)	++++	Yes
cg05341549 (*PIK3CD*)	0.008 (0.005, 0.010)	4.21E-04			0.0 (0.75)	++++	Yes
cg11075561 (*MAPK13^+^*)	0.010 (0.007, 0.014)	4.21E-04			33.4 (0.21)	++++	Yes
cg11585358 (*EPHX4^+^*)	0.007 (0.005, 0.009)	5.35E-04			31.3 (0.22)	++++	Yes
cg19905757 (*CORO2B*)	0.007 (0.005, 0.010)	5.99E-04			0.0 (0.97)	++++	Yes

Coefficients correspond to the expected change in placental DNA methylation per additional week of gestation after adjustment for placental cell-type proportions. Gene annotations were verified in the UCSC Genome Browser (hg19) with *^+^* indicating the closest gene. The direction of coefficients is ordered by cohort as follows: EARLI, ELGAN, Healthy Start, NHBCS.

The most notable associations observed after cell-type adjustment were for cg11650925 in females and cg12447744 in males, which each had the smallest p-values in the sex-specific meta-analyses (Table 4). For cg11650925, which is located within *GNG12-AS1*, a long non-coding RNA that is downstream of the imprinted gene *DIRAS3* [59], we observed a positive association between gestational age in females and placental methylation levels (coefficient = 0.012 per 1-week increase in gestational age, p-value = 8.95 × 10^−10^). It should be noted, however, that this association was unstable, as it did not persist when single cohorts were omitted from the meta-analysis. For cg12447744, which annotates to *MDM4*, higher gestational ages in males were associated with greater levels of placental methylation (coefficient = 0.008 per 1-week increase in gestational age, p-value = 2.06 × 10^−10^).

**Figure 5.**
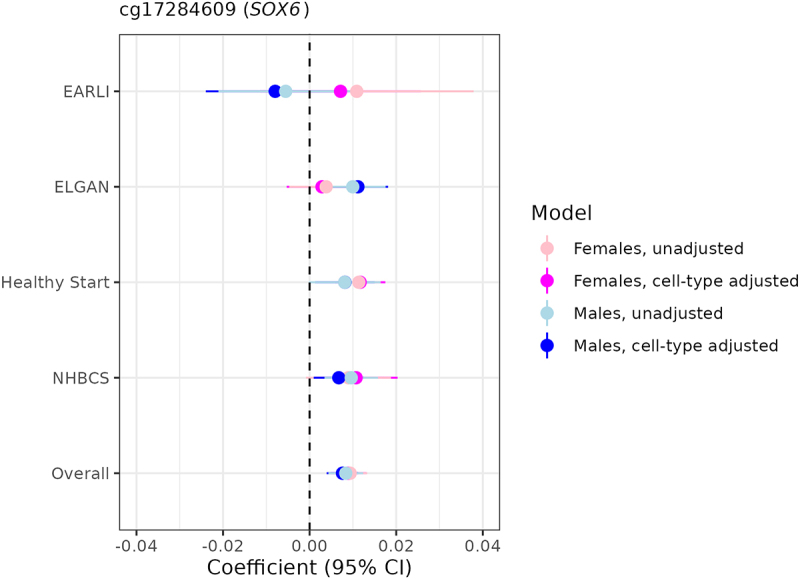
Sexually monomorphic associations of gestational age with placental methylation levels at cg17284609 (*SOX6*) before and after cell-type adjustment.

In terms of overlap between the two sexes, we found one probe that was FDR-significant among both females and males after adjusting for cell-type heterogeneity (Figure 2): cg17284609, which annotates to the promoter region of *SOX6*. Notably, this probe was also FDR-significant among both sexes in the unadjusted models (**Figure 5**) and survived all leave-one-out sensitivity analyses, indicating a profoundly robust relationship between increasing gestational age and higher levels of placental methylation at this specific site.

Forest plot depicting cohort-specific and meta-analysed associations of gestational age with placental methylation levels at cg17284609, the only probe that was FDR-significant in meta-analyses among both females and males before and after cell-type adjustment.

### Sex differences in associations of gestational age with placental CpG methylation

**Figure 6.**
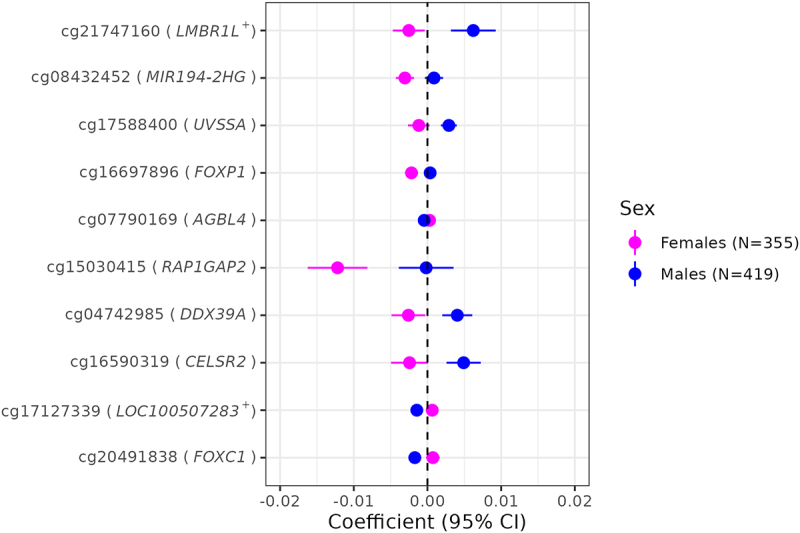
Sex-heterogeneous associations of gestational age with placental CpG methylation after cell-type adjustment.

Using the 880 FDR-significant CpGs from the cell-type adjusted models (55 CpGs in females and 826 in males, of which one overlapped), we performed two-sample Z-tests to test for sex-specific associations of gestational age with placental methylation. Those tests revealed that a total of 164 probes mapping to 116 genes showed evidence for sex-based heterogeneity in gestational age – placental CpG methylation associations at q < 0.05. We provide Z-test statistics with accompanying p-values and q-values for all 880 probes in **Supplemental Table 6** but highlight the top 10 most significant probes in Figure 6. Of the 164 sex-heterogenous probes, 108 (65.9%) had gestational age coefficients in opposing directions when comparing females and males. For instance, a 1-week increase in gestational age among females was inversely associated with placental methylation levels at cg21747160 (located near *LMBR1L*, Limb Development Membrane Protein 1 Like), whereas a positive association was observed among males (Figure 6).

Forest plot depicting the top 10 probes with methylation levels that differed most significantly between the two sexes. Gene annotations were verified in the UCSC Genome Browser (hg19) with + indicating the closest gene.

### Enrichment of distinct biological pathways

**Figure 7.**
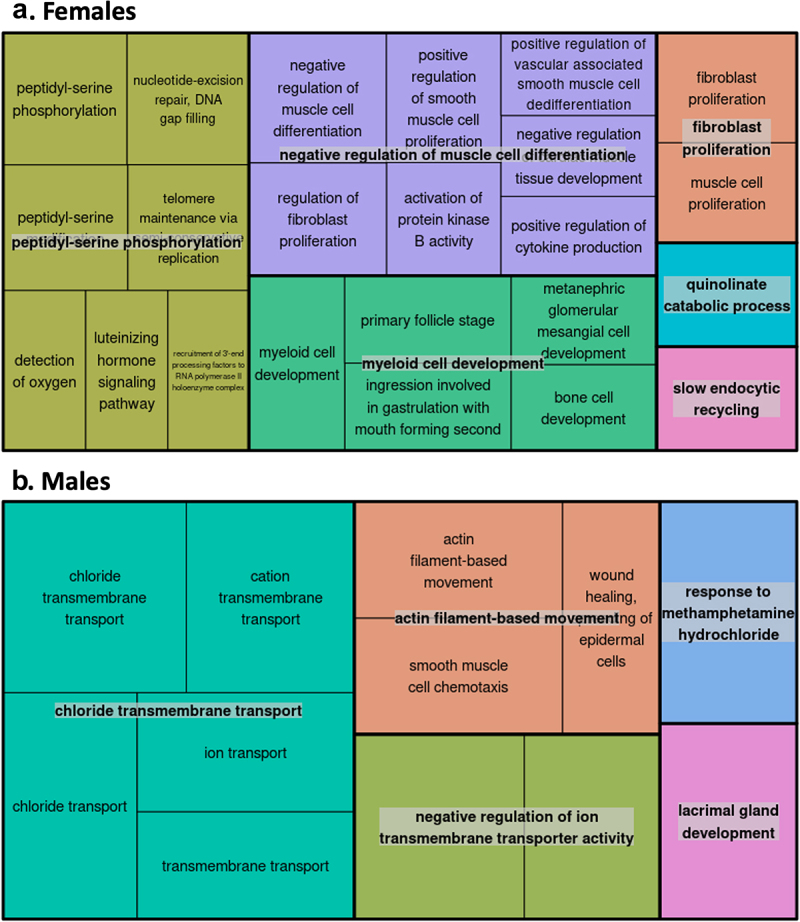
Gene Ontology (GO) enrichment analyses for the cell-type adjusted association of gestational age with placental CpG methylation among females and males.

Gene Ontology (GO) pathway analyses of the FDR-significant CpGs associated with gestational age after placental cell-type adjustment (annotating to 41 genes in females and 538 genes in males) revealed biological processes that differed between the two sexes. After being consolidated by REVIGO, top GO terms for females included cytokine production and myeloid cell development (Figure 7). In contrast, the top GO terms for males were primarily related to transmembrane transport (Figure 7).

**A**. The top 25 GO biological processes identified by enrichment p-value among female-associated CpG sites after undergoing REVIGO pruning. **B**. The top 25 GO biological processes identified by enrichment p-value among male-associated CpG sites after undergoing REVIGO pruning. GO term clusters are identified by rectangles with labels that reflect the least redundancy and highest degree of enrichment; colours represent related clustered GO terms, and the size of the rectangles is proportional to -log_10_(p-value for enrichment).

## Discussion

In this meta-analysis of four cohorts in the NIH ECHO programme, we found associations between gestational age and the placental DNA methylome that displayed striking differences by infant sex. In females, we identified 407 CpGs that were differentially methylated by gestational age; after accounting for placental cellular composition, this number was reduced to only 55 CpGs and most appeared unstable in leave-one-out analyses, indicating that cell-type heterogeneity largely explains female-specific associations. This finding may reflect placental cellular composition or levels of specific cell types promoting longer courses of gestation among females, which would be an interesting area for future research. We identified considerably more CpGs in males (794 CpGs in unadjusted models and 826 CpGs in cell-type–adjusted models, with the majority surviving leave-one-out analyses), suggesting a profound and direct relationship between increasing gestational age and placental methylation levels. Male-specific associations were generally positive in direction, an observation supported by a prior study of gestational age and DNA methylation in placental tissue [60]. Despite differences in study eligibility and participant characteristics across the EARLI, ELGAN, Healthy Start, and NHBCS cohorts, the findings were generally consistent, with the exception of results for females for whom cell – type adjustment produced unstable estimates that should be interpreted with caution [61]. Taken together, our findings emphasize that DNA methylation is an important mechanism underlying sex-based differences in the placenta, which may also be important for sex-specific health and developmental outcomes.

We identified a large number (164 CpGs) for which associations between gestational age and placental methylation levels appeared to be different between the two sexes. Those that differed most significantly by sex were located in or near the following genes: *LMBR1L, MIR194-2HG, UVSSA, FOXP1, AGBL4, RAP1GAP2, DDX39A, CELSR2*, and *FOXC1*. Two of these – namely *DDX39A* and *LMBR1L* – potentially contribute to immune responses and inflammation [62, 63], whereas *FOXP1* and *FOXC1* have been linked to preeclampsia and gestational diabetes, respectively [64, 65]. Based on our prior work, we anticipated that there would be some stark differences in the placental epigenome by sex; however, the exact mechanisms remain elusive [39, 66]. We speculate the CpGs displaying sex-divergent gestational age-methylation associations observed may be related to androgens produced by the foetus and placenta as early as the first trimester, fluctuating throughout the course of gestation, or may be related to other, non-hormonal sex-specific factors (e.g., anatomical differences) [67, 68]. Pathway analyses further revealed enrichment of distinct biological processes by sex. In females, myeloid cell development and cytokine production were among the top GO terms identified, highlighting pathways linked to the immune system and inflammatory responses. Enrichment among these pathways may explain why females mount a stronger immune response than males to various stimuli, including vaccines, pathogens, and autoantigens [69]. Sex-based epigenetic differences in immune responses may also explain disparities in later-in-life outcomes, as perinatal systemic inflammation has been linked to neurodevelopmental impairments such as autism and attention-deficit/hyperactivity disorder characterized by male predominance [70–72]. In this study, males displayed enrichment of GO terms related to transmembrane transport of solutes, including chlorides and cations. The translocation of solutes across biological membranes is of major physiologic importance. In the placenta specifically, molecular transport proteins in the syncytiotrophoblast and foetal capillary endothelium actively control the transport of a variety of compounds [73]. Both the under- and over-expression of such transporter proteins could affect the flux of key hormones, nutrients, and xenobiotics across the placental barrier [74, 75]. While we are unaware of any data showing that expression of transporter proteins in the placenta is sex-dependent [73], males appear to generally be more susceptible to the effects of prenatal chemical exposures [76], shedding light on one potential pathway underlying sex-based differences in pregnancy outcomes.

Prior research has examined the relationship between gestational age and autosomal DNA methylation in placental tissue without considering the role of foetal sex [26–28]. For instance, a 2019 study by Lee *et al*. derived three distinct placental ‘clocks’ to estimate ‘biological’ (as opposed to chronological) gestational age [26]. While the clocks are reported to have high accuracy in samples composed of both females and males, we observed limited overlap between the FDR-significant CpGs identified in this work and the CpGs composing the Robust Placental Clock (RPC), Control Placental Clock (CPC), and refined Robust Placental Clock (rRPC) [26]. One notable exception is cg05169312 (*GEMIN5*), our top hit among males in the unadjusted model that remained strongly associated with gestational age after accounting for placental cellular composition; we observed positive associations between gestational age and methylation levels for this CpG, consistent with its positive weights in each of the three Lee *et al*. clocks. The CpGs included in the clocks were selected by machine learning algorithms and therefore may prioritize predictive ability over biological meaning; however, our finding that many gestational age-related differences in placental CpG methylation levels differ by sex suggests there may be a need to move beyond ‘unisex’ placental epigenetic clocks and develop new clocks that are sex-specific.

Our study included data from four birth cohorts to provide foundational insights into the placental methylome but is not without limitations. To limit the burden on individual cohorts, each performed placental tissue sampling, DNA extraction, methylation interrogation, quality control, and normalization procedures according to their own existing protocols. Two cohorts (EARLI and NHBCS) measured placental methylation with the 450 K platform whereas the other two (ELGAN and Healthy Start) used the EPIC platform. By meta-analysing, we assumed measurements were comparable across the two platforms. In general, DNA methylation in placental tissue does show a high per-sample correlation between the arrays [77]. However, to be sure that our results were not misleading, we compared all CpGs we deemed as FDR-significant to a published list of probes that display absolute differences in percent methylation >50% in placental tissue across 450 K and EPIC; we found no overlap, supporting our use of the combined data. Some limitations of this study were the use of single placental biopsies to measure methylation profiles and the lack of standardization in sampling protocols across the cohorts. In addition, each cohort had its own eligibility criteria which contributed to distinct participant characteristics. For instance, ELGAN enrolled extremely preterm infants and thus was enriched for assisted reproduction and medically complicated pregnancies [78], whereas EARLI enrolled infants at high risk for autism. While these differences may have contributed to between-study heterogeneity, we found that only a small proportion of differentially methylated CpGs had I^2^ values at or above 50%, indicating that the results were largely consistent across the cohorts. We also performed leave-one-out sensitivity and found that, with the exception of females for whom cell – type adjustment rendered estimates unstable, the results were generally insensitive to the exclusion of any single cohort. Our approach assumed a linear relationship between DNA methylation and gestational age across the age ranges included in this study and limited the identification of relationships to those that are consistent across preterm and term placentas. Thus, we may have missed CpGs that exhibit non-linear associations within different windows of gestation. In future studies, it would be interesting to characterize how any observed relationships may differ between preterm and term placentas, although such an analysis would be more appropriate for cohorts spanning a wider range of gestational ages, or for meta-analyses that include multiple cohorts with greater overlap than this one, such that true differences could be distinguished from other unique cohort effects. There was also variability in how each cohort assessed gestational age. Measuring gestational age by foetal ultrasound, particularly when performed early in the first trimester, is considered to be the most accurate method for pregnancy dating [79]. While some of the included infants had ultrasound-based estimates, others had gestational ages calculated according to the date of the last menstrual period, a method prone to random error and an overestimation bias [80]. These inaccuracies may have introduced measurement error to our analyses but are expected to be small in magnitude as, on average, last menstrual period dating exceeds ultrasound-based estimates by fewer than 3 days [80]. Across the four cohorts, there were many complicated pregnancies and induced or caesarian section deliveries (224 of 355 females and 264 of 419 males, in total). Although pregnancy complications and obstetric interventions are associated with placental DNA methylation signatures [81, 82], our goal was simply to describe gestational age-related changes in the placental methylome separately for female and male infants. To that end, we did not control for pregnancy complications or interventions, because we did not want to make unverifiable assumptions regarding the temporal sequence of such factors with respect to gestational age, placental cellular composition, and placental CpG methylation [83]. Determining the chain of events will likely require non-human population study designs such as placental cell lines or animal models [84, 85]. Without placental mRNA data, we were unable to explore how differential CpG methylation correlated with gene expression, thus limiting our understanding of the functional relevance of gestational-age associated CpGs. Future studies of the placental methylome should consider using a systems biology approach to contextualize findings within molecular pathways. Another important point is that this meta-analysis only included infants assigned as female or male at birth and excluded a small number of samples for which the X and Y chromosome probe intensities did not match the reported sex. Because of our focus on the placenta, we also lacked information regarding gender identity, which develops in early childhood and may or may not agree with assigned sex. Although this work highlights female-male comparisons relating foetal growth and development to important placental epigenetic patterns, we acknowledge there is an urgent need to expand beyond binary sex classification to be more inclusive of intersex and transgender individuals. Finally, our analytic approach focused on identifying sex-based differences in gestational age – placental methylation associations, rather than identifying similarities in these associations between females and males. By analysing each sex separately, rather than pooled together, we may have missed some statistically weaker, but potentially biologically important relationships between gestational age and placental CpG methylation levels.

Our study has several strengths, including being the first to identify sex-heterogeneity in placental CpG methylation patterns related to gestational age. By combining data from four birth cohorts, we were able to increase the precision and generalizability of our findings. Importantly, through the inclusion of a preterm birth cohort as well as general birth cohorts, our study captured a wide range of gestational ages spanning 23–42 weeks. We were also able to control for placental cell-type heterogeneity using a relatively new reference-based approach [45]. Placental tissue is a complex mixture of trophoblasts, Hofbauer cells, endothelial cells, and stromal cells, which are epigenetically distinct and therefore need to be considered as a source of variability in epigenome-wide association studies [86]. Compared with reference-free cell-type deconvolution methods that have historically been used in studies of placental DNA methylation, the reference-based approach provides a more direct interpretation of placental cell composition. Its implementation allowed for comparisons of gestational age-placental methylation associations with and without cell-type adjustment, which can highlight differences between the sexes driven by placental cellular composition. Notably, we found that associations of gestational age with placental CpG methylation appear to be influenced by differences in cellular composition in females but not in males.

## Conclusions

In summary, we show that methylation levels at numerous CpG sites across the genome are associated with gestational age at birth in a sex-specific manner. Our findings highlight the importance of considering foetal sex in studies of the placenta, which may have utility for identifying the origins of sex differences in health that persist throughout life.

## Supplementary Material

Supplemental Material

## Data Availability

The individual-level datasets used for conducting EWASs in this manuscript are not publicly available because, per the NIH-approved ECHO Data Sharing Policy, ECHO-wide data have not yet been made available to the public for review/analysis. Requests to access the individual-level datasets should be directed to the ECHO Data Analysis Center, ECHO-DAC@rti.org. However, the EWAS summary statistics from each cohort that were used to conduct subsequent meta-analyses are available through the UNC Dataverse at https://doi.org/10.15139/S3/K73DN9. All software and programs used to conduct these analyses are freely available and are cited in the manuscript. All code used to generate our results is available on the UNC Dataverse at https://doi.org/10.15139/S3/EEB75X.
