# Supplementary material for: Sex-based differences in placental DNA methylation profiles related to gestational age: an NIH ECHO meta-analysis
Source: Epigenetics. 2023 Feb 25;18(1):2179726. doi: 10.1080/15592294.2023.2179726 (PMC9980626; doi:10.1080/15592294.2023.2179726)
Supplement: Supplemental Material [file KEPI_A_2179726_SM3040.zip › Supplementary files/Supplementary captions.docx]

**ADDITIONAL FILES**

**Supplemental Material**

**Supplemental Figure 1:** Flow diagram depicting inclusion/exclusion criteria for CpG probes and placental samples within each cohort and for meta-analysis

**Supplemental Figure 2:** Quantile-quantile (QQ) plot showing -log_10_ transformed p-values from each EWAS before and after BACON-correction

**Supplemental Figure 3:** Hierarchically clustered heatmap of *planet*-estimated placental cell-type proportions annotated by sex and gestational age within EARLI (n=91)

**Supplemental Figure 4:** Hierarchically clustered heatmap of *planet*-estimated placental cell-type proportions annotated by sex and gestational age within ELGAN (n=242)

**Supplemental Figure 5:** Hierarchically clustered heatmap of *planet*-estimated placental cell-type proportions annotated by sex and gestational age within Healthy Start (n=98)

**Supplemental Figure 6:** Hierarchically clustered heatmap of *planet*-estimated placental cell-type proportions annotated by sex and gestational age within NHBCS (n=343)

**Supplemental Table 1:** Participant characteristics across the cohorts included in the meta-analysis

**Supplemental Table 2:** Correction of batch effects by EARLI, ELGAN, Healthy Start, and NHBCS

**Supplemental Table 3:** A summary of inflation before and after BACON-correction

**Supplemental Table 4:** Meta-analysis results of gestational age without placental cell-type adjustment for probes that were FDR-significant in both females and males from EARLI, ELGAN, Healthy Start, and NHBCS

**Supplemental Table 5:** Meta-analysis results for the association between gestational age and placental CpG methylation among females and males from EARLI, ELGAN, Healthy Start, and NHBCS

**Supplemental Table 6:** Meta-analysis results for the association between gestational age and placental CpG methylation after cell-type adjustment among females and males from EARLI, ELGAN, Healthy Start, and NHBCS. Two-sample Z-tests were performed to test for heterogeneity of gestational age coefficients by sex on probes that were FDR-significant among either females or males.
